# The Oomycete *Pythium oligandrum* Can Suppress and Kill the Causative Agents of Dermatophytoses

**DOI:** 10.1007/s11046-018-0277-2

**Published:** 2018-07-02

**Authors:** Alena Gabrielová, Karel Mencl, Martin Suchánek, Radim Klimeš, Vít Hubka, Miroslav Kolařík

**Affiliations:** 1 Laboratory of Fungal Genetics and Metabolism, Institute of Microbiology, Czech Academy of Sciences, Vídeňská 1083, 14220 Praha 4, Czech Republic; 2 Laboratory of Medical Mycology, Department of Microbiology, Pardubice Regional Hospital, 56024 Pardubice, Czech Republic; 3 Bio Agens Research and Development – BARD, Rýznerova 150, 25262 Únětice, Czech Republic; 4 Biopreparáty spol. s. r.o., Rýznerova 150, 25262 Únětice, Czech Republic

**Keywords:** *Pythium oligandrum*, Dermatophytes, Mycoparasitism, Aggressivity genes, *Trichophyton*, *Microsporum*

## Abstract

*Pythium oligandrum* (Oomycota) is known for its strong mycoparasitism against more than 50 fungal and oomycete species. However, the ability of this oomycete to suppress and kill the causal agents of dermatophytoses is yet to be studied. We provide a complex study of the interactions between *P. oligandrum* and dermatophytes representing all species dominating in the developed countries. We assessed its biocidal potential by performing growth tests, on both solid and liquid cultivation media and by conducting a pilot clinical study. In addition, we studied the molecular background of mycoparasitism using expression profiles of genes responsible for the attack on the side of *P. oligandrum* and the stress response on the side of *Microsporum canis*. We showed that dermatophytes are efficiently suppressed or killed by *P. oligandrum* in the artificial conditions of cultivations media between 48 and 72 h after first contact. Significant intra- and interspecies variability was noted. Of the 69 patients included in the acute regimen study, symptoms were completely eliminated in 79% of the patients suffering from foot odour, hyperhidrosis disappeared in 67% of cases, clinical signs of dermatomycoses could no longer be observed in 83% of patients, and 15% of persons were relieved of symptoms of onychomycosis. Our investigations provide clear evidence that the oomycete is able to recognize and kill dermatophytes using recognition mechanisms that resemble those described in oomycetes attacking fungi infecting plants, albeit with some notable differences.

**Electronic supplementary material:**

The online version of this article (10.1007/s11046-018-0277-2) contains supplementary material, which is available to authorized users.

## Introduction

Dermatophytic fungi of the genera *Trichophyton, Nannizzia, Microsporum* and *Epidermophyton* cause infections of keratinized structures such as the skin, hairs and nails of healthy individuals [1]. Although infections by dermatophytes are usually not life-threatening, they are widespread and difficult to eliminate completely [2]. According to World Health Organization statistics, the global prevalence of dermatophytoses is approaching 20–25%, making it one of the most frequent infectious diseases, with treatment costs estimated at half a billion dollars annually [3, 4]. Dermatophytes are grouped ecologically according to their habitat as being either anthropophilic (human-associated), zoophilic (animal-associated) or geophilic (soil-dwelling). Virulence factors of dermatophytes remained unknown until recently, when comparative genome analyses revealed candidate genes possibly involved in the infection process [5–7]. Three different classes of genes and their products are cited most often as critical factors: proteases secreted to degrade skin, kinases involved in signalling necessary for the interaction between the host and the fungus and LysM adhesins that appear to bind to surface carbohydrates of dermatophytes and mask them from the immune response of the host. These latter factors, in particular, appear to be responsible for the poor recognition of dermatophytes by keratinocytes and macrophages, and the subsequent inhibition of an effective immune response of infected organisms against dermatophytes.

Considering the low efficiency of chemical antifungals against dermatophytes [8, 9], their elimination using biological defence means would appear as an attractive alternative. However, the biological enemy will have to offer universal and safe elimination mechanisms, considering the physiological and etiological variability among individual dermatophytes. *Pythium oligandrum* is a non-pathogenic soil-inhabiting peronosporomycete (oomycete) colonizing the root ecosystems of many crop species [10]. This microorganism exhibits strong mycoparasitism against more than 50 fungal and oomycete species, including some of its relatives [11]. To become such an efficient parasite, *P. oligandrum* has developed a number of traits allowing it to recognize, engage and destroy target fungi [12, 13] and it is assumed that it acquired the parasitism genes from the three eukaryotic kingdoms and from bacteria [14]. Hydrolytic enzymes, such as chitinases, cellulases, proteases and glucanases, secreted by *P. oligandrum* are often cited as molecular tools critical for their mycoparasitic success [10, 15]. Competition for space and nutrients are other mechanisms used by *P. oligandrum* for biological control [16]. The unique possibilities of this microorganism have been used extensively for the protection of plants from fungi [11, 16–19]. In addition, *P. oligandrum* has found practical use in human and veterinary medicine for the elimination of dermatophytes [19, 20]. Despite the practical medical observations cited above, the exact cellular and molecular mechanisms behind the elimination of the causal agents of dermatophytoses have not been investigated.

Here we provide a systemic study of the interactions between *P. oligandrum* and dermatophytes representing all ecological groups and species that dominate in developed countries [21, 22]. We assessed its biocidal potential by performing growth tests, both on solid and on liquid cultivation media, and by conducting a clinical study. We studied the molecular background of mycoparasitism using expression profiles of genes responsible for the attack on the side of *P. oligandrum* and the stress response on the side of *Microsporum canis*. Our investigations provide clear evidence that the oomycete is able to recognize and kill dermatophytes using recognition mechanisms that resemble those described in oomycetes attacking fungi infecting plants, albeit with some notable differences.

## Materials and Methods

### Microbial Strains and Media

The M1 strain of *P. oligandrum* was provided by the company Biopreparáty, Ltd (Únětice, Czech Republic) and corresponds to strain ATCC 38472. This oomycete was isolated from sugar beet [18, 23]. Ten different species and 23 different strains of dermatophytes were obtained from the CCF collection (Culture Collection of Fungi, Charles University, Prague, Czech R.), from the CCM collection (Czech Collection of Microorganisms, Masaryk University, Brno, Czech R.) or from the working collection kept at the Institute of Microbiology of the Czech Academy of Sciences. Competition tests on solid media were carried out for the most common species of dermatophytes, including one strain of *Epidermophyton floccosum*, one strain of *M. canis,* two strains of *Nannizzia fulva* (syn. *Microsporum fulvum*), two strains of *N. gypsea* (*Microsporum gypseum*), two strains of *N. persicolor* (syn. *Microsporum persicolor*), five strains of *Trichophyton benhamiae* (syn. *Arthroderma benhamiae*), one strain of *Trichophyton erinacei,* four strains of *Trichophyton interdigitale,* three strains of *Trichophyton rubrum* and one strain of *Trichophyton tonsurans* (Online Resource 1). The strain *M. canis* CCM 8353 was used for the gene expression study. The identity of all strains was verified using ITS rDNA barcode sequence and PCR fingerprinting comparisons with reference strain according to Hubka et al. [24].

### Interaction Studies on Plates

The interaction between the dermatophytes and *P. oligandrum* was done on malt extract agar (MEA, malt extract, 20 g/l, d-glucose, 20 g/l, peptone, 1 g/l, agar 20 g/l) and potato dextrose agar (PDA, HI Media) incubated at 25 °C in the dark. First, the dermatophytes under examination were inoculated on one side of the plate and allowed to grow for 3–10 days, producing colonies 20–25 mm in size. Thereafter, the agar block with *P. oligandrum* was placed onto the opposite side of the Petri dish, and the continuation of the growth of the dermatophyte and *Pythium* were evaluated every 2 days until 10 days. The measured parameter was the percentage occupancy of plates calculated as the distance of the front of the growing microorganism from the edge of the Petri dish (in mm) divided by the diameter of the plate (80 mm) and multiplied by 100. Each experiment was performed in triplicate on each of the media used. At the end of the experiment, the viability of the tested dermatophytes was also evaluated. An agar block (1 × 1 cm) was cut from the interaction zone, where both organisms were visibly present, and transferred to Czapek-Dox agar (CDA, sucrose 30 g/l, agar 20 g/l, NaN0_3_ 3 g/l, K_2_HPO_4_ 1 g/l, MgSO_4_ 0.5 g/l, KCl, 0.5 g/l, Fe_2_(SO_4_)_3_ 0.01 g/l, pH 6.5), which enables the growth of the dermatophytes but not of *P. oligandrum.*

### Interaction Studies in Suspensions

The evaluation of the *P. oligandrum* fungicidal effect was according to EU Standards [ČSN EN 1275: 2006: Chemical disinfectants and antiseptics—quantitative suspension test for the evaluation of basic fungicidal or basic yeasticidal activity of chemical disinfectants and antiseptics—test method and requirements (phase 1)] and was conducted by the test laboratory Chemila, spol. s r.o. (Hodonín, Czech R., accredited as the test laboratory No. 1273 by Czech Accreditation Institute according to the norm ČSN EN ISO/IEC 17025). The strains used were *Trichophyton rubrum* strain 584/2017, *Trichophyton interdigitale* CCM 8377 and *M. canis* CCM 8353 (Online Resource 1). Test suspensions of dermatophytes were prepared by washing the spores with 0.05% polysorbate 80 in water, gentle shaking with the glass beads and filtration through a fritted filter with porosity 40–100 μm. The tested preparation of *P. oligandrum* (batch No. 060217.3, BARD s.r.o.) was resuspended in the distilled water to get 1% suspension with concentration ranging from 100 to 200 CFU/ml and activated for 30 min at 20 °C. During the suspension test, 0.5 ml of dermatophyte spore suspensions (density of 3.04 × 10^6^/ml for *T. rubrum*, 0.88 × 10^6^/ml for *T. interdigitale* and 0.26 × 10^6^/ml for *M. canis*) was mixed with 0.5 ml of the *P. oligandrum* suspension and incubated at 20 ± 1 °C for 1 h, 24 h and 48 h. Subsequently, tenfold serial dilutions were prepared from the incubated suspensions and number of CFU was evaluated by the cultivation on the Sabouraud agar for 7–10 days at 25 °C. Results are expressed as logarithmic microbial viability reduction for each test microorganism designated as log *R* (reduction of vitality). The log *R* is calculated based on the formula log *R* = log *N*_0_ − log *N*_E_, which accounts the concentration (CFU/ml) of the dermatophyte at the beginning (*N*_0_) and at the end of the contact time (*N*_E_) with *P. oligandrum*.

### Gene Expression Profiles on Agar Plates and in the Liquid Suspension

The three genes connected with mycoparasitism on the side of *P. oligandrum* [10, 15] and aggressivity and stress response on the side of dermatophyte [6] were selected for the understanding of the molecular mechanisms standing behind interaction of both organisms (Table 1). For gene expression profiles on agar plates, samples of both the dermatophyte and *P. oligandrum* were taken by cutting the agar blocks (4 × 8 mm) from the interaction zone and zone with pure *P. oligandrum* or dermatophyte as is shown in Fig. 4. The lower part of the block containing the agar was removed, and the upper part with the mycelium was used for DNA and RNA extraction using the protocol of Berendzen et al. [25]. Gene expression profiling started from day 4 (just before the physical contact of both microbes) and proceeded until day 7. For gene expression profiles in liquid suspension, conidia of *M. canis* CCM 8353 were prepared and counted using the method described by Saunte et al. [26]. A suspension of *P. oligandrum* was obtained using a liquid culture and counted on the basis of all reproductive forms, sporangia, zoospores and oospores [23]. To initiate the interaction experiment, 4 ml of MEA (without agar) medium was mixed with 0.5 ml of *M. canis* conidia (5 × 10^6^ conidia/ml) and 0.5 ml of *P. oligandrum* (as the sum of reproductive forms, 5 × 10^6^ cells/ml) in six-well plastic plates (BioTech, Czech Republic) and incubated at 30 °C. In control experiments, one of the microorganisms was omitted and replaced by a pure medium. From each well, 50 µl of the liquid medium was collected in 6-h intervals for subsequent culturing on CDA plates for viability testing and gene expression profiling. Good aeration and homogeneity of the sample were assured by its frequent agitation.

**Table 1:** The primers used for the assessing of the expression profiles of *Pythium oligandrum* and *Microsporum canis*

Protein name^a^	Protein function	Primer name	Primer sequence (5′-3′)	References
*Pythium oligandrum*				
Cellulase (endo-β-1,4-glucanase) (POCELL)	Cell wall lysis and reorganisation (42)	POCELLFW	AGAACAAGTCTGGCGACGAG	This study, designed based on EST clone EV244394
		POCELLRE	GTTCGGACGACTGTTCCACT	
Endo-β-1,3-glucanase (putative) (POENDO)	Cell wall lysis and reorganisation, sporangia development (42, 43)	POEN13FW	AACTACGACTTGCGTCAGGG	This study, designed based on EST clone EV245189
		POEN13RE	ACGTTCTTGGTGATCGTGCT	
Small tyrosine-rich proteins (POSTRU)	Oospore formation (34)	POST15FW	GTGCCTATGGCTACGACGAC	[36]
		POST15RE	GTGGTGCTTGTGGTGCTTC	
β-Tubulin	Microtubule formation	POTUBAFW	GATGTCGTGCCAAAGGATGTC	[36]
		POTUBARE	CGAAGGTGGCTGGTAGTTGATAC	
Glyceraldehyde-3-phosphate	Glycolysis	POGAPDFW	GGACATCATCCGTAAGGCGT	[28]^b^
		POGADPRE	TGAAGAGATCACGGAGCACG	
*Microsporum canis*				
LysM protein (MCLYSM)	Cell wall surface masking (14)	MCLYSMFW	ATACCGGACTGGGAACTGGA	This study, designed based on sequence XM_003174875
		MCLYSMRE	CGGCCTATCGTACGTCTTCC	
Keratin-specific metalloproteinase (MCMETA)	Keratin degradation (14)	MCMETAFW	CTCTCCACGAGTTCACCCAC	This study, designed based on sequence XM_002846474
		MCMETARE	GCAGCCGACGTAGATAGCAT	
Ca^2+/^calmodulin-dependent protein kinase (MCCAMK)	Cell signalling regulating growth and stress response (14)	MCCAMKFW	AAACTGTGGGAAAAAGCGGC	This study, designed based on sequence XM_002847552
		MCCAMKRE	TGGCACATCTTGTCACTCCC	
β-Tubulin	Microtubule formation	MCBETUFW	CACCTTCGTCGGAAACTCCA	This study, designed based on sequence XM_002848601
		MCBETURE	CATCTCGTCCATACCCTCGC	
Glyceraldehyde-3-phosphate	Glycolysis	MCGAPDFW	CACTTGAAGGGAGGTGCCTA	This study, designed based on sequence XM_002848601
		MCGAPDRE	CTGCATCTCGGGCTTGTAGT	

^a^Protein abbreviation used in this study is shown

^b^The original study used *Pythium splendens*, which had the priming sites identical with *P. oligandrum* (GenBank accession: LSAJ01000098.1)

The primers were used based on the cited literature or designed with the Primer-Blast tool (http://ncbi.nlm.nih.gov/tools/primer-blast) (Table 1). Specificity of the PCR primers was confirmed by the sequencing of their PCR products. All DNA/RNA amplifications were performed using the CFX Connect Real-Time PCR Detection System operated using the CFX Manager™ Software ver. 3.0 (BioRad) as described previously [27]. Briefly, reverse transcription reactions were performed in Hard-Shell^®^ 96-Well PCR plates (BioRad) in the total reaction volume of 10 µl composed of 5 µl of the individual, stabilized RNA samples and 5 µl of the reverse transcription master mix A + B (Generi Biotech, Czech Republic). Each plate was sealed and incubated at 42 °C for 60 min. Thereafter, the reaction mixtures were diluted 50× using RNAse-free water according to the instructions of the manufacturer. The qPCR amplifications were performed in Hard-Shell^®^ 96-Well PCR plates in the total reaction volume of 10 µl composed of 4 µl of diluted cDNA (see above), 1 µl of the primer mixture containing 5 µM of each of the corresponding forward and reverse primer, and 5 µl of SsoAdvanced Universal SYBR^®^ Green Supermix (BioRad). The plates were placed into the CFX Connect Real-Time PCR System (BioRad) operated using CFX Manager™ Software. The cycling parameters were: 95 °C for 3 min followed by 50 cycles of 95 °C for 10 s and 60 °C for 30 s. The data were evaluated using the 2^−ΔΔCt^ (Livak) method as described in the Real-Time PCR Application Guide (BioRad). The expression of inducible genes was related to the expression of two constitutive reference genes for beta tubulin and glyceraldehyde-3-phosphate dehydrogenase, which expression varies within the range ± 50% under the range of tested conditions [28, 29]. Gene expression on the interaction plate was corrected based on gene expression on the control plate containing the single microorganisms under examination [27].

### Clinical Study

This study is a retrospective clinical trial conducted at the Department of Dermatology and Venerology of the Pardubice Regional Hospital in Pardubice between 1 June 2007 and 1 June 2014. No randomization of patients was performed. Informed consent was obtained from all individual participants included in the study. Patients were mostly outpatients included in the study based on the following criteria. For the study of acute patients (*n* = 69), these criteria were: clinical symptoms of foot mycoses confirmed by either microscopic observation or a positive microbial cultivation test. For the recurrent infection study (*n* = 29), the criteria were: recurring problems with tinea interdigitalis infection at least twice yearly, an acute attack of tinea interdigitalis present upon entering the study and confirmation of foot mycosis by both microscopy and cultivation. No patients had to be excluded from the study on the basis of their age or clinical status. From the set of 29 patients with recurrent infection, 16 had an acute attack of tinea interdigitalis at least twice yearly, 9 of them three times a year, 3 patients stated four attacks annually, and 1 person suffered problems with the disease continuously. In acute patients, the profile of the causative agents revealed using cultivation was consistent with published data, with *T. rubrum* and *T. interdigitale* (8) representing two of the by far most common species (Online Resource 2). In patients with recurrent infection, the most common causal agent was *T. rubrum* (72% of patients), sometimes in combination with candidiasis. *Pythium oligandrum* was applied in the form of the cosmetic product Biodeur ^®^ (Bio Agens Research and Development, BARD s.r.o., Únětice, Czech R.) which is composed of *P. oligandrum* M1 dried spores (> 2 × 10^5^ oospores/g) stored in the presence of a silica desiccative and dried millet (*Panicum miliaceum*), which provides the substrate for the revitalization of the microorganism. For the foot baths, 1 g of the Biodeur ^®^ was reconstituted in 2–3 l of tap water warmed to 34 °C. Patients’ feet were washed in this solution for 30 min and allowed to dry spontaneously. For acute infections, the foot bath was applied on two alternate days in three consecutive weeks, and the medical evaluation was performed 1 month after the last bath. The protocol for recurrent tinea interdigitalis patients was based on the initial bolus, identical to the acute situation with additional applications in weeks 5, 7 and 10, followed by maintenance applications 6 weeks apart. As an additional preventive measure, shoe spraying by Biodeur ^®^ suspension was applied twice per week in weeks 1–5, followed by once per week sprays in weeks 6–9, and additional sprays every second week thereafter. In the study of patients with recurrent tinea interdigitalis, continuous monitoring in the form of regular checks followed, the time of the cessation of the disease was evaluated based on clinical evaluation supplemented by mycological evaluation by microscopy and cultivation.

## Results

### Patterns and Kinetics of Interaction Between *P. oligandrum* and Dermatophytes on Plates and in the Suspension

**Fig. 1:**
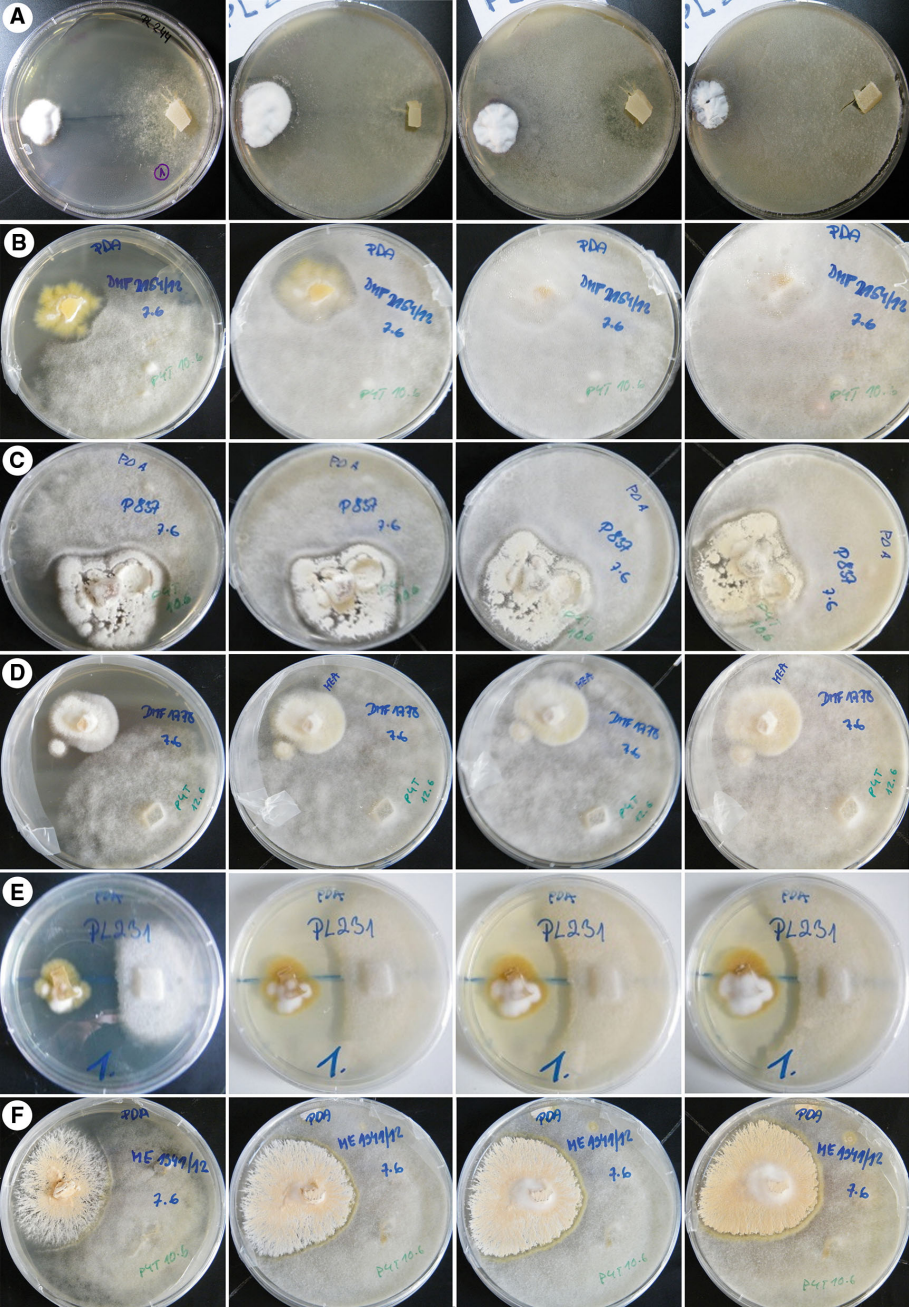
Examples of the time course of direct interactions representing all five interaction patterns. Type I—exponential single phase of the ascending type. **a** *Trichophyton rubrum* CCF 4933. **b** *Trichophyton benhamiae* CCF 4918. Type II—exponential two-phase pattern of the ascending type. **c** *Trichophyton erinacei* CCF 4472. Type III—exponential single phase with an ascending and a descending phase**. d**. *Nannizzia persicolor* CCF 4542. **e** *Epidermophyton floccosum* PL 231. Type IV—two-phase pattern with an ascending and a descending phase. **f.** *Nannizzia gypsea* CCF 4626. The photograph was taken on days 4, 6, 8 and 10 of the experiment, ordered sequentially from left to right

**Fig. 2:**
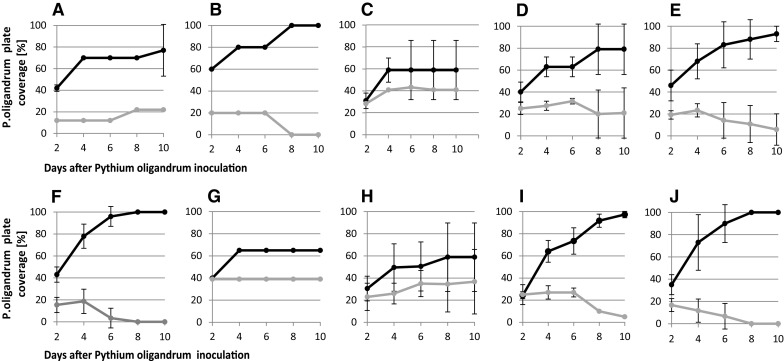
Examples of the time course of the elimination of dermatophytes (gray line) by *Pythium oligandrum* (black line) on the MEA cultivation medium. **a** *Epidermophyton floccosum.* **b** *Microsporum canis.* **c** *Nannizzia fulva* **d** *Nannizzia gypsea.* **e** *Nannizzia persicolor.* **f** *Trichophyton benhamiae.* **g** *Trichophyton erinacei.* **h** *Trichophyton interdigitale.* **i** *Trichophyton rubrum.* **j** *Trichophyton tonsurans.* Error bars represent the standard deviation counted from all strains and Petri dishes of the particular dermatophyte species

The three types of growth pattern scenarios were observed. Firstly, the growth of *P. oligandrum* over the dermatophyte was observed in case of *M. canis*, *N. persicolor, T. benhamiae, T. rubrum* and *T. tonsurans* (Fig. 1). Concerning the kinetics of the interaction, *T. benhamiae, T. rubrum* or *T. tonsurans*, got rapidly overgrown by the oomycete. In *M. canis*, the curve is biphasic, indicating a certain degree adaptation to *Pythium* in the initial stage (Fig. 2). In other species, the interaction started with the formation of a contact inhibition zone of various intensity, varying from wide (*N. fulva*, *T. erinacei, T. interdigitale* and *E. floccosum*) to narrow (*N. gypsea*) (Fig. 1). This interaction occurred at the level of substrate mycelium and was later followed by the production of aerial mycelium by *P. oligandrum*, which overgrew the dermatophyte species. The last category of dermatophytes includes *N. fulva* and some strains of *N. gypsea, N. persicolor* and *T. interdigitale* that were able to adapt to the attack mounted by *Pythium* and, eventually, to stop its action.

**Fig. 3:**
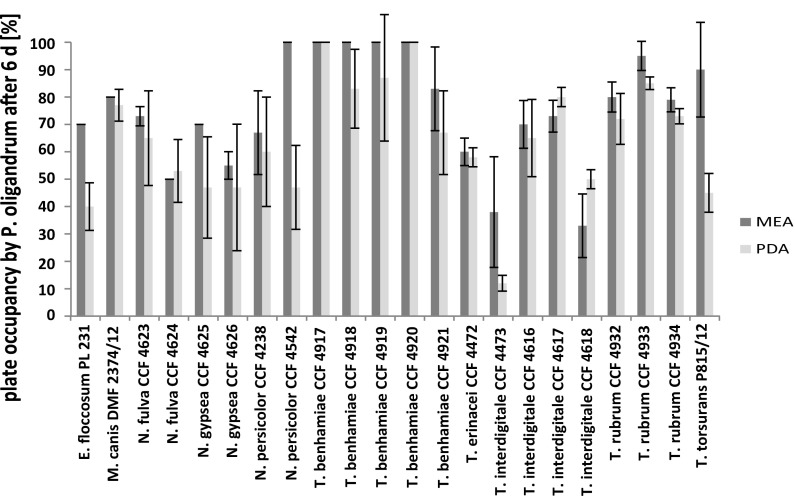
Effects of the medium and strain on the competition between *Pythium oligandrum* and dermatophytes after 6 days of the experiment. Error bars represent the standard deviation counted from all strains and Petri dishes of the particular dermatophyte species

The competitive ability of *P. oligandrum* on MEA was generally better than on PDA, with the exception of *N. fulva* and certain strains of *T. interdigitale*. Excluding species from which only a single strain was available, notable intraspecies variability was found in *N*. *fulva*, *N. gypsea* and *N. persicolor* (Fig. 3).

Viability tests were conducted after the dermatophyte overgrown by *P.* *oligandrum* was transferred to CDA medium allowing the growth of the dermatophyte, but not of the *Pythium.* Using this approach, it could be shown that the dermatophytes were actually dead after their interaction with the *Pythium.*

Under the conditions of the liquid culture, the 3–4 log viability reduction of the target dermatophyte after 48 h was observed (Table 2).

**Table 2:** Reduction of vitality of three dermatophytes after exposure of *P. oligandrum* in liquid culture

Test organism	Contact time (h)	Log *R* (vitality reduction)
*T. interdigitale*	1	0.38
	24	≥ 3.64
	48	≥ 3.64
*T. rubrum*	1	0.14
	24	2.69
	48	≥ 4.18
*M. canis*	1	0
	24	≥ 3.11
	48	≥ 3.11

### Gene Expression Profiles and Viability During the Elimination of *Microsporum canis* on the Plate

**Fig. 4:**
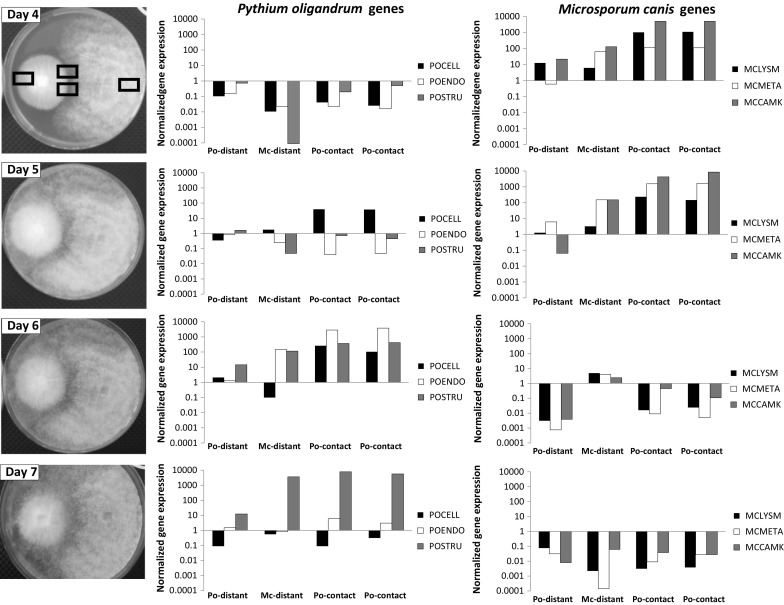
Gene expression profiles during the interaction of *Pythium oligandrum* with the dermatophyte *Microsporum canis.* An agar block with *Pythium oligandrum* was added on day 3 onto a Petri dish with a well-grown dermatophyte. Photodocumentation and gene expression profiling started from day 4 and proceeded until day 7. For *Pythium oligandrum*, we examined the expression of genes coding for cellulase (POCELL), endo-β-1,3-glucanase (POENDO) and the tyrosine-rich structural protein (POSTRU), whereas for *Microsporum canis* we followed the expression of genes for the LysM adhesion/masking protein (MCLYSM), metalloproteinase (MCMETA) and Ca-dependent kinase (MCCAMK). The results are shown at logarithmic scale; each bar shows the average value for three independent experiments

The results regarding the expression of inducible aggressivity genes in *P. oligandrum* and *M. canis* are detailed in Fig. 4. Only expression relative to beta tubulin is shown since the expression relative to glyceraldehyde-3-phosphate dehydrogenase provided very similar data (not shown). Concerning the oomycete, on day 4 the expression of all genes was very low compared to the control situation. After the direct contact on day 5, the genes for the critical digestion enzyme cellulose (POCELL) got switched on, followed by the expression of cell wall lysing endo-β-1,3-glucanase (POENDO) and sporulation marker POSTRU with the peak on the day 6. On the day 7, only the POSTRU expression persisted, when the oomycete had most probably exhausted the available nutrients and switched its metabolism to the sporulation mode.

In case of dermatophyte, there was considerable upregulation of all genes before the contact of both taxa on the day 4 and after the contact on the day 5. Then, on day 6, the biological antagonism between the oomycete and the dermatophytes was decided for the oomycete. This was documented by the dramatic cessation of the expression of all analysed genes, with only negligible amounts of transcripts being detected.

### Gene Expression Profiles and Viability During the Elimination of *Microsporum canis* Conidia in Suspension

**Fig. 5:**
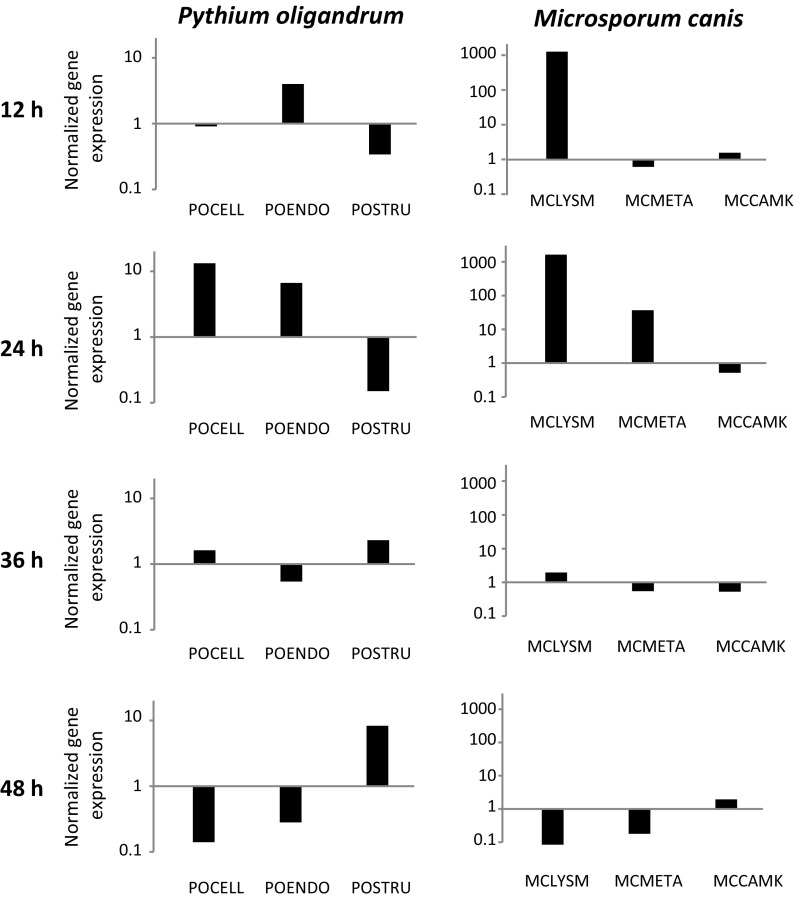
Gene expression profiles and viability during the interaction of *Pythium oligandrum* with the dermatophyte *Microsporum canis* in suspension. For *Pythium oligandrum,* we examined the expression of genes coding for cellulase (POCELL), endo-β-1,3-glucanase (POENDO) and the tyrosine-rich structural protein (POSTRU) whereas while for *Microsporum canis*, we followed the expression of genes for the LysM effector/masking protein (MCLYSM), metalloproteinase (MCMETA) and Ca-dependent kinase (MCCAMK). The results are shown at logarithmic scale; each bar shows the averaged value for three independent experiments

The gene expression profiles under the suspension conditions showed the same pattern as observed in the plate experiment (Figs. 4, 5). The notable difference was in the expression of the POENDO gene which preceded the expression of the POCELL gene, and in the normalized expression levels were generally much lower. Furthermore, while the dermatophyte retained considerable overexpression of the MCLYSM and MCMETA genes, we observed very little expression of the MCCAMK gene. The cultivation experiment shows that the development of the dermatophyte could be suppressed completely within 60 h (Online Resource 3).

### Clinical Efficacy of *Pythium oligandrum* in Patients with Tinea Pedis

**Fig. 6:**
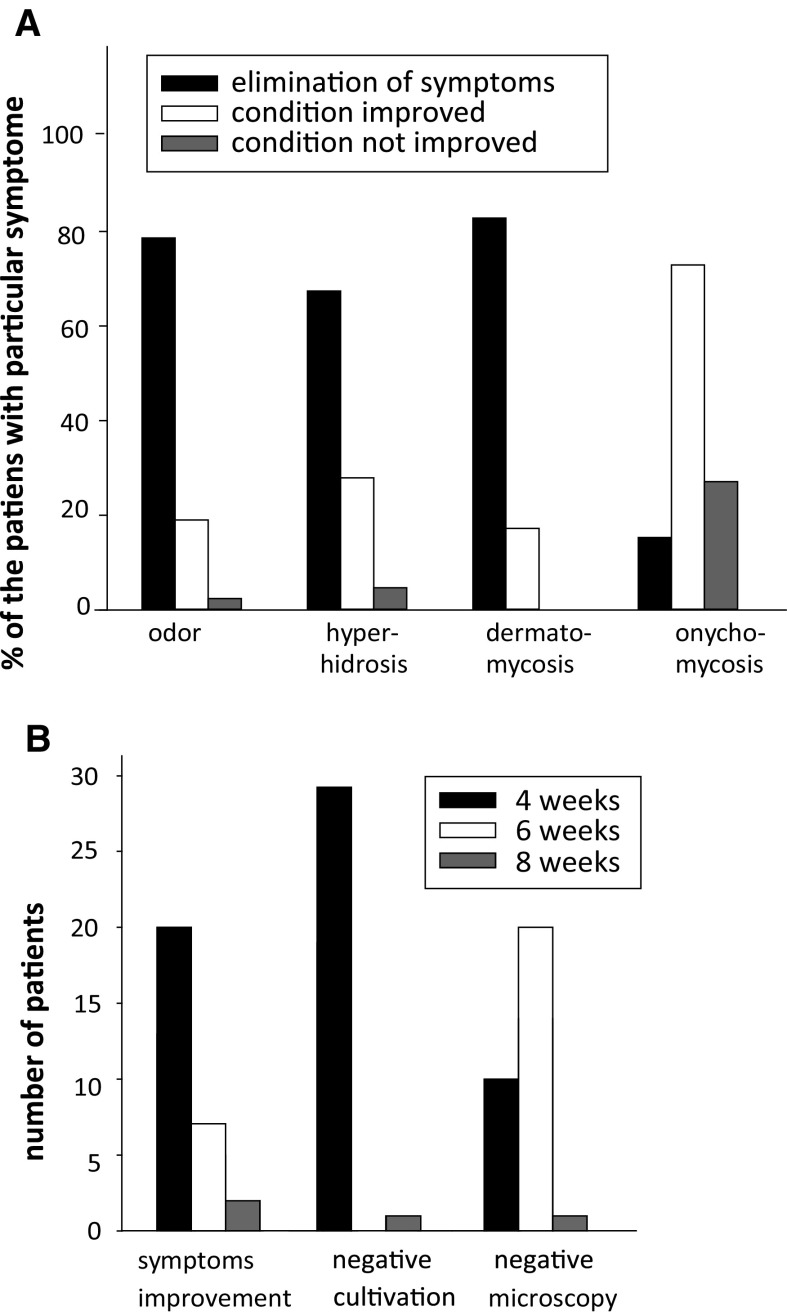
Efficacy of cosmetic products containing the oomycete *Pythium oligandrum* against symptoms of foot mycoses. **a** Elimination of individual symptoms in patients with acute patients. **b** Elimination of individual symptoms in patients with recurrent dermatophytoses

The efficacy of relief of foot mycoses symptoms is summarized in Fig. 6. Of the 69 patients included in the acute regimen study, 42 had odour symptoms, 43 exhibited hyperhidrosis, 58 had dermatomycosis, and 59 suffered from onychomycosis. Symptoms were completely eliminated in 79% of the patients suffering from foot odour, hyperhidrosis disappeared in 67% of cases, clinical signs of dermatomycoses could no longer be observed in 83% of patients, and 15% of persons were relieved of symptoms of onychomycosis (Fig. 6b). In patients with recurring infections, in 28 (97%) of all 29 patients, clinical symptoms disappeared within 6 weeks after the first application. Concerning the long-term follow-up of all 29 patients, only three patients that finished the 12-month application protocol had a single further episode of tinea interdigitalis within the next 3 months. The application of the biological cosmetic product containing *P. oligandrum* was well tolerated. We did not observe a single episode of an allergic reaction or any other side effect in either the acute or the recurrent group of patients (Fig. 6c).

## Discussion

The ability of *P. oligandrum* to parasite on other fungi and oomycetes has been known since the forties of the last century [30]. However, this important aspect of the biology of this peronosporomycete has so far been analysed only in relation to its ability to provide protection against plant pathogens (reviewed in [10, 31]). We used three approaches to perform our investigations: direct observations of interactions in dual cultures, molecular analyses of gene expression profiles of both interacting microorganism and clinical tests in real conditions. In the cases of dermatophytes investigated on the Petri dishes, we could distinguish at least four patterns of interaction (Fig. 2): pattern I, defined as an exponential single phase of the ascending type (*T. benhamiae, T. rubrum* and *T. tonsurans*); pattern II, defined as an exponential two-phase pattern of the ascending type (*M. canis* and *T. erinacei*); pattern III, defined as an exponential single phase with an ascending and a descending phase (*E. floccosum N. fulva* and *N. persicolor*); and, lastly, pattern IV, defined as a two-phase pattern with an ascending and a descending phase (e.g. *N. gypsea* and *T. interdigitale*). It may be assumed that such patterns might reflect features of the more detailed mechanisms of mutual interactions controlled by consecutive waves of mutual recognition of the target dermatophyte by the oomycete, or consecutive waves of diffusion of soluble molecular factors (the detailed nature of which is yet to be ascertained).

In general, the results of the biological fight between the oomycete *P. oligandrum* and the target fungus is expected to be both species- and medium- (environment-) specific, and the presented observations confirm this (Fig. 3). Nevertheless, the entire group, including *T. rubrum*, a dominant dermatophyte species, is mostly killed and eliminated by *P. oligandrum* with high efficiency. The high efficiency of the biological elimination of the target dermatophyte species is further corroborated by the suspension interaction results, where the log suppression reached a 3–4 log reduction within 48 h (Table 2) that is required for chemical biocides [32]. Such uniformly high efficiency of killing is not common in other groups of fungi, where more extensive variations between the efficiency of killing individual fungi are observed [30].

Our molecular analyses provided an additional understanding of the molecular nature of the antagonism between the oomycete and the dermatophytes. It was interesting to observe that while in the case of *P. oligandrum* direct contact with its prey appeared to initiate very little changes in the gene expression profiles, the dermatophytes must have been informed about the presence of the biological enemy long before the actual contact (dramatic gene upregulation on day 4, Fig. 4). We hypothesize that volatile or diffusible compounds produced by *P. oligandrum* (reviewed in [16]) can mediate this kind of early response in the attacked fungus.

In case of the gene expression profiles of *P. oligandrum,* the sequence of gene upregulation could be explained based on our knowledge of the biological functions of the individual gene markers. Both cellulase (POCELL) and endo-β-1,3-glucanase (POENDO) belong to the group of glycohydrolase, which are able to digest the cell wall of the attacked fungus [33, 34]. However, remodelling of the own cell wall of the oomycete allows the endoglucanase to feature also as an important marker of sporulation [35]. The tyrosine-rich structural protein (POSTRU) has recently been identified as one of the most specific markers of sporulation and is thus expressed during the late stages [36]. Indeed, the sequence of gene expression in the plate experiment started with the upregulation of cellulase on day 5 of the experiment, especially in the samples taken from proximal areas of the contact zone between the oomycete and the dermatophyte. All three followed genes of the oomycete were significantly upregulated on day 6 of the experiment, albeit the cellulase only in the contact areas (Fig. 4). On day 7 of the experiment, sporulation of the oomycete was expected, which was supported by the persistent upregulation of the tyrosine-rich structural protein (Fig. 4). The time sequence of gene expression of the oomycete during the suspension experiment was somewhat different, reflecting different cellular populations and a different environment (Fig. 5). Here, the endoglucanase gene was expressed earlier compared to the plate experiments at 12 h after mixing, while both cellulase and endoglucanase genes are highly expressed at 24 h after mixing. The tyrosine-rich structural protein is expressed only at 48 h after mixing, when the sporulation phase occurred under the solution experimental conditions (Fig. 5).

The three dermatophyte genes followed in the present study are typical aggressivity and stress related genes [6]. LysM cell surface effector (MCLYSM) represents an important surface effector and masking protein that covers the surface of dermatophytes to block access of soluble protein recognition factors of the immune system. This mechanism is acting in plant-pathogenic fungi and in the response of dermatophytes to the human immune system [6]. We have shown that dermatophytes use this stress-induced pathway also in their response to mycoparasitism. Metalloprotease (MCMETA) is the principal digestion enzyme that allows dermatophytes to digest the skin protein keratin and thus mediates both the attachment of fungi to skin structures and their nutrition. Ca-dependent kinase (MCCAMK) is one of the most important signalling enzymes triggering and orchestrating the response of the dermatophytes. These genes were used because the genes that are upregulated during the biological struggle of the dermatophytes with the oomycete were not known. They were all switched very early on, showing that the dermatophytes were able to sense the enemy and react, and remained switched on until the moment of the death (Fig. 4). We again noticed some principal differences between the plate and the suspension experiment. In the plate experiment, the upregulation was much more dramatic and concerned all three followed genes on day 4 and day 5 of the experiment. In suspension, only the MCLYSM gene was upregulated at 12 h and accompanied by MCMETA at 24 h (Fig. 5).

In 2002, Mencl described the use of the cosmetic biopreparation Biodeur, containing a fermented millet substrate with a surface growth of *P. oligandrum* for the suppression of the hidrotic feet syndrome (foot sweating) and interdigital mycoses [19]. This author reported an effect 1 month after its application on patients having infections with *T. rubrum, T. interdigitale*, and other dermatophytes and yeasts. In patients subjected to that study, there was 78.6% elimination of odour symptoms, 67.4% elimination of the hidrotic symptom and 82.8% elimination of the dermatophyte infection, evidenced by the absence of the dermatophyte upon the microbial cultivation.

Our clinical results from the initial study can be viewed as remarkable, considering that dermatophytoses in humans are notoriously difficult to treat, and often recurring. We noticed a high efficiency of the cosmetic product containing oospores of the oomycete *P. oligandrum* in the elimination of clinical signs of dermatophytosis, odour symptoms and hyperhidrosis (83–67% of patients, *n* = 69). Onychomycoses are known to take much longer to resolve compared to other symptoms. Indeed, although notable recovery from interdigital damage could be observed as soon as 20 days after the first application of the biological products, as long as 9 months was needed to observe definitive signs of recovery in cases of onychomycosis (not shown).

In conclusion, the present study demonstrates the ability of the oomycete *P. oligandrum* to suppress or eliminate dermatophytes, emphasizing its efficiency against a broad spectrum encompassing virtually all clinically important species and demonstrating a susceptibility profile that has not been observed for the classes or species of fungi targeted by this oomycete. By using of viable *P. oligandrum* propagules, it was possible to prove that their suppressing effect on dermatophytes starts within hours of the mutual encounter and interaction. Our detailed description of such an aggressive type of parasitism provides a scope for the practical use of the findings presented here.

## Electronic supplementary material

Below is the link to the electronic supplementary material.

**Online Resource 1.** The list of dermatophytes used in the study (DOCX 18 kb)

**Online Resource 2.** Incidence of dermatophytes, other fungi and yeast in acute patients (DOCX 105 kb)

**Online Resource 3**. The viability of *Microsporum canis* during the co-cultivation with *Pythium oligandrum* in the liquid suspension (experiment from the Fig. 5) (DOCX 13 kb)
