# Supplementary material for: The Oomycete Pythium oligandrum Can Suppress and Kill the Causative Agents of Dermatophytoses
Source: Mycopathologia. 2018 Jul 2;183(5):751–64. doi: 10.1007/s11046-018-0277-2 (PMC6156753; doi:10.1007/s11046-018-0277-2)
Supplement: Supplementary file 1 — Online Resource 1. The list of dermatophytes used in the study (DOCX 18 kb) [file 11046_2018_277_MOESM1_ESM.docx]

**Supporting material to**

**The oomycete *Pythium oligandrum* can suppress and kill the causative agents of dermatophytoses**

By Alena Gabrielová, Karel Mencl, Martin Suchánek, Radim Klimeš, Vít Hubka, Miroslav Kolařík

**Supporting Table 1 –** The list of the dermatophytes used in the study. All strains, except of *Microsporum canis* CCM 8353 were used in the competitive assays on the solid media. The liquid culture based qPCR assays were done on the *M. canis* CCM 8353.

^a^ – FP

| **Strains used in the competitive assays on the solid media** | | | |
| --- | --- | --- | --- |
| **Species** | **Strain** | **Origin** | **ITS rDNA acc. nos. ^a^** |
| *Epidermophyton floccosum* | PL 231 = PL 1605/12 | tinea pedis, sole skin of 29-year-old male, Czech Rep. | FP |
| *Microsporum canis* | DMF 2374/12 | tinea faciei, 14-year-old girl, Czech Rep. | LT897803 |
| *Nannizzia fulva* | CCF 4623 (ME 1236/12) | tinea corporis, arm skin of 63-year-old female, Czech Rep. | LT897804 |
| *Nannizzia fulva* | CCF 4624 (P245/11) | tinea corporis, forearm skin of 46-year-old female, Czech Rep. | HG518408 |
| *Nannizzia gypsea* | CCF 4626 (ME 1341/12) | tinea corporis, instep skin of 28-year-old female, Czech Rep. | LN878968 |
| *Nannizzia gypsea* | CCF 4625 (P255/12) | tinea corporis, arm skin, 51-year-old female, Czech Rep. | LT897805 |
| *Nannizzia persicolor* | CCF 4542 (DMF 1778/12) | tinea corporis, dorsum of the hand of 45-year-old male, Czech Rep. | LT897806 |
| *Nannizzia persicolor* | CCF 4238 (KVK 2124/11) | fingernail of 12-year-old girl, Czech Rep. | LT897807 |
| *Trichophyton benhamiae* | CCF 4918 (DMF 2154/12) | tinea corporis, forearm skin of 18-year-old male, Czech Rep. | LT897800 |
| *Trichophyton benhamiae* | CCF 4919 (DMF 1049/12) | tinea corporis, forearm skin of 8-year-old girl, Czech Rep. | FP |
| *Trichophyton benhamiae* | CCF 4920 (DMF 2158/11) | tinea faciei, 56-year-old female, Czech Rep. | LT897801 |
| *Trichophyton benhamiae* | CCF 4917 (DMF 953/12) | onychomycosis, toenail of 68-year-old female, Czech Rep. | LN874020 |
| *Trichophyton benhamiae* | CCF 4921 (DMF 3148/11) | tinea corporis, forearm skin of 38-year-old female, Czech Rep. | LT897802 |
| *Trichophyton erinacei* | CCF 4472 (P837/12) | tinea corporis, neck skin of 16-year-old female, Czech Rep. | LN614531 |
| *Trichophyton interdigitale* | CCF 4616 (DMF 2525/12) | tinea corporis, back skin of 47-year-old female, Czech Rep. | LT897808 |
| *Trichophyton interdigitale* | CCF 4618 (DMF 2477/12) | tinea corporis, trunk skin of 50-year-old female, Czech Rep. | FP |
| *Trichophyton interdigitale* | CCF 4617 (DMF 3857/12) | tinea corporis, arm skin of 26-year-old female, Czech Rep. | FP |
| *Trichophyton interdigitale* | CCF 4473 (P852/12) | tinea corporis, thigh skin of 14-year-old female, Czech Rep. | LN736306 |
| *Trichophyton rubrum* | CCF 4933 (PL 244 = 2608/12**)** | tinea corporis, 3-year-old boy, Czech Rep. | LT897809 |
| *Trichophyton rubrum* | CCF 4932 (D410/13) | onychomycosis, toenail of 47-year-old male, Czech Rep. | LT897810 |
| *Trichophyton rubrum* | CCF 4934 (10020/13) | tinea corporis, buttock skin of 39-year-old male, Czech Rep. | LT897811 |
| *Trichophyton rubrum* | 584/2017 | not provided, strain deposited at the Pardubice Regional Hospital in Pardubice, Czech Republic |  |
| *Trichophyton tonsurans* | P815/12 | tinea corporis, arm skin of 23-year-old male, Czech Rep. | LT897812 |

| **Strains used in the competitive assays in the liquid media** | | | |
| --- | --- | --- | --- |
| **Species** | **Strain** | **Origin** | **ITS rDNA acc. nos. ^a^** |
| *Trichophyton rubrum* | 584/2017 | not provided, strain deposited at the Pardubice Regional Hospital in Pardubice, Czech Republic |  |
| *Trichophyton interdigitale* | CCM 8377 | clinical specimen (human tinea pedis); = DSM 4870, ATCC 9533 |  |
| *Microsporum canis* | CCM 8353 | dermatomycosis of skin of man, Czech. Rep |  |

| **Strains used in the liquid culture based qPCR assays** | | | |
| --- | --- | --- | --- |
| **Species** | **Strain** | **Origin** | **ITS rDNA acc. nos. ^a^** |
| *Microsporum canis* | CCM 8353 | dermatomycosis of skin of man, Czech. Rep |  |
