# Supplementary figures and images for: The Oomycete Pythium oligandrum Can Suppress and Kill the Causative Agents of Dermatophytoses
Source: Mycopathologia. 2018 Jul 2;183(5):751–64. doi: 10.1007/s11046-018-0277-2 (PMC6156753; doi:10.1007/s11046-018-0277-2)

**Online Resource 2.** Incidence of dermatophytes, other fungi and yeast in acute patients.


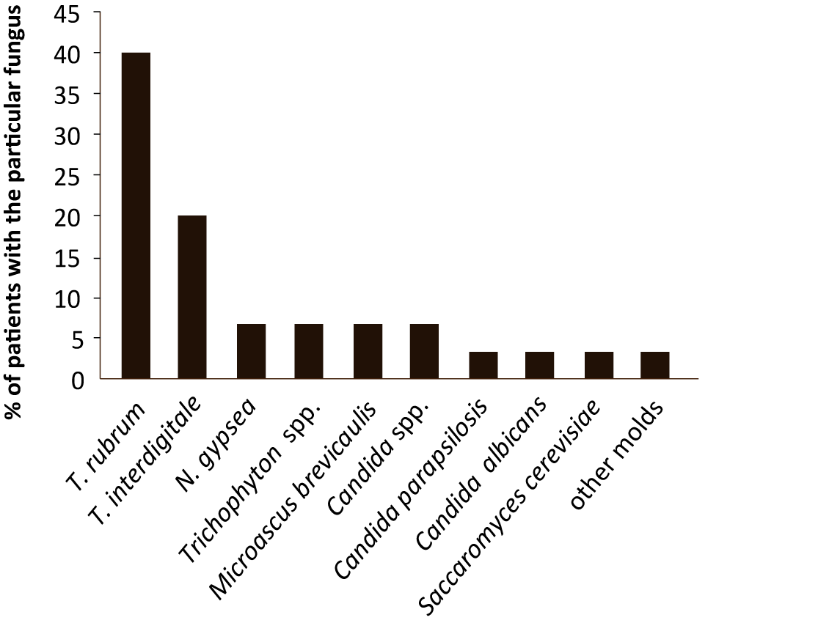

Supplement: Supplementary file 2 — Online Resource 2. Incidence of dermatophytes, other fungi and yeast in acute patients (DOCX 105 kb) [file 11046_2018_277_MOESM2_ESM.docx]
