# Supplementary material for: The Oomycete Pythium oligandrum Can Suppress and Kill the Causative Agents of Dermatophytoses
Source: Mycopathologia. 2018 Jul 2;183(5):751–64. doi: 10.1007/s11046-018-0277-2 (PMC6156753; doi:10.1007/s11046-018-0277-2)
Supplement: Supplementary file 3 — Online Resource 3. The viability of Microsporum canis during the co-cultivation with Pythium oligandrum in the liquid suspension (experiment from the Fig. 5) (DOCX 13 kb) [file 11046_2018_277_MOESM3_ESM.docx]

| **Hrs after mixing** | **Number of viable colonies/ml ^a^** | **Log suppression per 24 h** |
| --- | --- | --- |
| 0 | 1125000 ± 30000 |  |
| 6 | 1345000 ± 35000 |  |
| 12 | 650000 ± 23000 |  |
| 18 | 230000 ± 13500 |  |
| 24 | 34200 ± 2340 | 1.51 |
| 30 | 22000 ± 2300 |  |
| 36 | 9840 ± 980 |  |
| 42 | 3250 ± 230 |  |
| 48 | 840 ± 120 | 1.61 |
| 54 | 230 ± 34 |  |
| 60 | 34 ± 6 |  |
| 66 | n.d. | n.d. |
| 72 | n.d. | n.a. |

**Online Resource 3.** Kinetics of killing of the dermatophyte *Microsporum canis* by the oomycete *Pythium oligandrum* during the suspension interaction (experiment from the Fig. 5). During this experiment, 10^6^ of spores (microconidia) of the dermatophyte *Microsporum canis* was mixed with growing *Pythium oligandrum* containing 10^5^ viable cells.

^a^ – average from three experiments ± S.D., n.d. – not detected
